# Sleep duration and mortality in Korean adults: a population-based prospective cohort study

**DOI:** 10.1186/s12889-020-09720-3

**Published:** 2020-10-28

**Authors:** Sohyeon Kwon, Hyeyoung Lee, Jong-Tae Lee, Min-Jeong Shin, Sangbum Choi, Hannah Oh

**Affiliations:** 1 Interdisciplinary Program in Precision Public Health, Department of Public Health Science, Graduate School of Korea University, 145 Anam-ro, Hana Science Building B, Seongbuk-gu, Seoul, Republic of Korea; 2 Department of Statistics, Graduate School of Korea University, 145 Anam-ro, Woodang Hall, Seongbuk-gu, Seoul, Republic of Korea; 3 Department of Health Policy and Management, College of Health Science, Korea University, Seoul, Republic of Korea; 4 Department of Biosystems and Biomedical Sciences, College of Health Science, Korea University, Seoul, Republic of Korea

**Keywords:** Sleep, Death, Mortality, Asian, Race, Cohort study

## Abstract

**Background:**

Increasing evidence suggests that sleep duration is associated with risks of various diseases including type 2 diabetes, cardiovascular disease (CVD), and certain types of cancer. However, the relationship with mortality is not clear, particularly in non-European populations. In this study, we investigated the association between sleep duration and mortality in a population-based prospective cohort of Korean adults.

**Methods:**

This analysis included 34,264 participants (14,704 men and 19,560 women) of the Korea National Health and Nutrition Examination Survey (KNHANES) 2007–2013 who agreed to mortality follow-up through December 31, 2016. Sleep duration was self-reported at baseline and was categorized into four groups: ≤4, 5–6, 7–8, and ≥ 9 h/day. Cox proportional hazards models were performed to estimate hazard ratios (HR) and 95% confidence intervals (CI) for the associations with mortality (all-cause as well as CVD- and cancer-specific), adjusting for potential confounders.

**Results:**

During up to 9.5 years of follow-up, we identified a total of 1028 deaths. We observed the lowest mortality at 5–6 h/day sleep. Compared with 7–8 h/day of sleep, short (≤4 h/day) and long (≥9 h/day) sleep were associated with a 1.05-fold (95% CI = 0.79–1.39) and 1.47-fold (95% CI = 1.15–1.87) higher all-cause mortality, respectively. After additional adjustment for self-rated health, the positive association with short sleep disappeared (HR = 0.99, 95% CI = 0.75–1.32) and the association with long sleep was slightly attenuated (HR = 1.38, 95% CI = 1.08–1.76). Long sleep was also nonsignificantly positively associated with both cancer-mortality (HR = 1.30, 95% CI = 0.86–1.98) and CVD-mortality (HR = 1.27, 95% CI = 0.73–2.21). There was no statistically significant evidence for nonlinearity in the relationships between sleep duration and mortality (all-cause as well as CVD- and cancer-specific). Effect modification by age, sex, education, and occupation were not statistically significant.

**Conclusions:**

Our findings suggest that long sleep duration is associated with an increased all-cause mortality in Korean adults.

## Background

Sleep is one of the essential activities of human life that has an influence on immune system [1–3], inflammation [4], metabolic profiles [5], and energy expenditure [6]. Studies have shown that poor quality and quantity of sleep may have negative health effects. Both short and long sleep are associated with increased risks of type 2 diabetes [7], cardiovascular disease (CVD) [8], and certain types of cancer [9, 10]. Short sleep, in particular, has been associated with alteration in glucose metabolism, upregulation of appetite [6], and increased levels of C-reactive protein (a pro-inflammatory marker) [11] and carotid intima-media thickness (atherosclerosis indicators) [12]. Through these relationships with intermediate endpoints and biomarkers, sleep duration is likely to be associated with mortality.

To date, many studies have investigated the relationship between sleep duration and mortality. However, the results are mixed and the exact shape of the relationship is unclear. Some studies reported a U-shaped relationship with all-cause and disease-specific mortality [13, 14] showing increased mortality at both < 7 and > 8 h/day of sleep [15–17], while others found a positive association with prolonged sleep only [18]. The possible explanations for the inconsistent findings are the inadequate control for confounding by preexisting diseases [19, 20], as the failure to control for these biases may lead to misleading results. Further, previous studies that investigated the sleep duration-mortality relationship were primarily conducted in Western countries, limiting generalizability to other populations such as Asians. Because sleep patterns may be influenced by social-cultural factors and personal lifestyle factors such as TV watching and physical activity, sleep patterns, including sleep duration, and their associations with mortality are likely to vary by geographic region [21, 22]. In this study, we examined the relationship between sleep duration and mortality (all-cause, as well as cancer- and CVD-specific) in a population-based prospective cohort of Korean men and women. We accounted for confounding by careful adjustment for potential confounders including demographic characteristics, lifestyle factors, and self-rated health at baseline. To identify the shape of the relationship and the optimal sleep duration associated with the lowest mortality risk, this study further investigated a non-linear dose-response relationship between sleep duration and mortality. Lastly, to identify the most susceptible populations, we also examined the effect modification by demographic and socioeconomic factors.

## Methods

### Study population

The Korea National Health and Nutrition Examination Survey (KNHANES) is a series of nationwide cross-sectional survey which has been administered by the Korea Centers for Disease Control and Prevention (KCDC) and the Korean Ministry of Health and Welfare in 1998, 2001, 2005, and annually since 2007. The target population of the survey is comprised of nationally representative non-institutionalized civilians aged 1 year and older. All subjects were selected using a stratified, multistage probability cluster sampling based on sex, age, and geographic area [23]. Each survey was conducted using similar designs and sampling methods and included three parts: health examinations, a health interview, and a nutrition survey. Trained staff members in the mobile examination centers conducted the health interview, following the standardized KNHANES protocols [23]. The health interview included the collection of information on demographic characteristics, medical history, self-rated health, and lifestyle (including sleep duration). All participants provided informed consent and the survey was approved by the Ethics Committee of the KCDC.

This analysis included the KNHANES 2007–2013 participants who agreed to mortality follow-up through December 31, 2016. Among 58,423 KNHANES 2007–2013 participants, 53,420 (91%) agreed to mortality follow up. Baseline characteristics were similar between participants who agreed vs. did not agree to mortality follow up, except that those who agreed had slightly higher levels of education. Among 53,420 participants with mortality data, we excluded those who were at age ≤ 19 years (*n =* 13,452), had missing or implausible values of sleep duration (≤2 or ≥ 22 h/day) (*n =* 1344), were currently pregnant (*n =* 201), and had unknown or extreme values of body mass index (BMI; < 15 or > 50 kg/m^2^) (*n =* 155). To reduce confounding by preexisting diseases, we further excluded participants who reported to have a history (“have ever had a diagnosis” or “currently have”) of cancer (*n =* 1163), stroke (*n =* 779), or heart disease (*n =* 851) at baseline, those with missing information or reported to have “very poor” self-rated health (*n =* 1118) at baseline, and those who died during the first year of follow-up (*n* = 93). The final analytic population included a total of 34,264 participants (14,704 men and 19,560 women).

### Assessment of sleep duration

During health interview, participants were asked to report the average hours (in integer) of sleep per day during the past year. Based on this information, we created a categorical sleep duration variable of four levels (≤4, 5–6, 7–8, and ≥ 9 h/day) and used 7–8 h/day, the most common category, as the reference level.

### Mortality ascertainment

The vital records (e.g., death status, date and causes of death) of study participants were ascertained through the linkage to death certificates and medical records. Information on primary causes of death were indicated in the *International Classification of Diseases and Related Health Problems* (ICD-10) code. The endpoints of our analyses were all-cause mortality, CVD mortality (including stroke and heart disease; I00-I99), and cancer mortality (C00-D48).

### Covariate assessment

Weight and height were measured at physical examination. BMI was calculated by weight (kg) divided by height squared (m^2^). Given a nonlinear relationship with mortality, BMI was categorized into four groups based on the Asia-Pacific obesity classification cutpoints [24]: < 18.5 (underweight), 18.5–22.9 (normal), 23.0–24.9 (overweight) and ≥ 25.0 kg/m^2^ (obesity). Demographic characteristics and lifestyle information was assessed via health interview. Participants’ reports on alcohol drinking history (never, at least once), frequency (< 1 time/month, 1 time/month, 2–4 times/month, 2–3 times/week, 4 times/week), and the usual drinking amount (1–2, 3–4, 5–6, 7–9, ≥10 glasses) were used to calculate the number of glasses per day of alcohol drinking. Smoking status (never, former, current) was defined using the questionnaire that asked “How many cigarettes have you smoked in your lifetime?” and “Are you currently smoking?” Current smokers were defined as those who have smoked ≥100 cigarettes (five packs) in lifetime and reported as a current smoker. To rule out recent initiators, we used ≥100 cigarettes criteria to define current smokers. Participants were asked to report the average frequency (0–7 days/week) and duration (h/day) they spent on walking, moderate- and vigorous-intensity physical activities. We calculated metabolic equivalent task (MET)-h/wk. of total physical activity for each participant by assigning 3 METs to walking, 4 METs to moderate-intensity activities, and 8 METs to vigorous-intensity activities. According to the National Physical Activity Guideline, we categorized participants into those meeting (≥10 MET-hr/wk) vs. not meeting the guideline (< 10 MET-hr/wk). Metabolic syndrome was assessed through health interview and physical examination. Metabolic syndrome was defined as meeting three or more of the following conditions: 1) waist circumference ≥ 85 cm for women and ≥ 90 cm for men, 2) fasting triglyceride level ≥ 150 mg/dL or on hyperlipidemia medication, 3) HDL cholesterol level < 40 mg/dL for men and < 50 mg/dL for women, or on hyperlipidemia medication, 4) fasting blood glucose level ≥ 100 mg/dL, on treatment (insulin injection or oral hypoglycemic agent), or reported a diagnosis of diabetes, 5) systolic blood pressure ≥ 130 mmHg, diastolic blood pressure ≥ 85 mmHg, or on hypertension medication.

### Statistical analysis

We used Cox proportional hazards models to estimate hazard ratios (HRs) and 95% confidence intervals (CIs) for all-cause mortality, CVD mortality, and cancer mortality by sleep duration categories. We used age (in month) as the time metameter, stratifying by the calendar year of survey [25]. All analyses were accounted for sampling weights and complex survey design (cluster, strata) [23, 26]. In disease-specific mortality analyses, we counted the disease-specific death as an outcome event and censored the deaths from other causes, assuming that those who were censored have the same disease-specific mortality as those who continued to be followed. We ascertained that the proportional-hazards assumption was not violated by using log-log plots to check that the curves of sleep duration categories were parallel and by confirming that the interaction with the time variable was not statistically significant (*p* = 0.64). Multivariable models included potential confounders: demographic characteristics (sex, marital status, education, occupation, household income, region) and lifestyle factors (BMI, smoking status, physical activity, alcohol drinking). For each covariate, missing values were less than 3%. We imputed the missing values using predictive mean matching method [27] for continuous variables and using the most frequent category for categorical variables. For missing values of metabolic syndrome, we created a missing category. In secondary analysis, we additionally adjusted for self-rated health to assess the influence of confounding by subclinical disease. To assess the extent to which the association can be explained by metabolic syndrome, we further adjusted for metabolic syndrome (a potential mediator) [28]. In sensitivity analyses, we excluded deaths that occurred during the first 2 years of follow-up and performed a 2-year time-lagged analysis to assess the influence of subclinical disease at baseline. Because the influence of baseline subclinical disease may be greater with shorter follow-up, we restricted the analyses to those with ≥5 years of follow-up in a separate sensitivity analysis. To examine the nonlinear shape of the relationship between sleep duration and mortality, we also performed restricted cubic spline analyses with five knots at the 5th, 35th, 50th, 65th, 95th percentiles of sleep duration. We tested for nonlinearity using the likelihood ratio test for comparing restricted cubic spline models vs. linear models. To examine the presence of effect modification, we stratified the analyses by age, sex, education, and occupation and tested for statistical interaction using Wald test for product terms. All statistical analyses were performed using SAS 9.4 (Cary, NC, USA) and R 3.6.1 (Vienna, Austria).

## Results

During up to 9.5 years of follow up (mean: 6.3 years), 34,264 participants contributed a total of 181,440 person-years and 1028 total deaths (216 CVD deaths and 352 cancer deaths). Table 1 shows the baseline characteristics of study participants according to sleep duration categories. Compared with 7–8 h/day of sleep, participants with ≤4 and ≥9 h/day sleep were more likely to be female, older, and unemployed and to have poor self-rated health, lower household income, and completed less than high school education.

**Table 1:** Baseline characteristics of 34,264 study participants by sleep duration

Characteristics	Sleep Duration, h/day			
	≤4	5–6	7–8	≥9
**N**	1388	12,730	17,589	2557
**Weighted N**^**a**^	7,446,267	83,398,188	117,845,933	16,421,072
	**Weighted**^**a**^ **percentage or mean (SD)**			
**Sex** ^**b**^				
Male, %	37.7	52.1	51.2	43.1
**Age (years)**^**b**^				
20–49, %	34.1	64.0	69.9	66.4
50–59, %	19.4	18.7	16.9	13.4
60–69, %	19.1	10.1	8.2	8.7
≥70, %	27.3	7.3	5.0	11.5
**Education**^**b**^				
Less than high school, %	59.4	27.7	22.5	31.9
High school, %	25.5	38.7	41.5	43.4
College or higher, %	15.0	33.7	36.0	24.6
**Occupation**^**b**^				
Non-physical labor, %	10.7	26.4	25.4	13.7
Physical labor, %	40.7	42.8	40.2	36.0
Unemployed, %	48.6	30.8	34.4	50.3
**Household income**^**b**^				
Quartile 1 (lowest), %	33.3	13.3	12.1	20.0
Quartile 2, %	25.2	26.0	25.6	26.9
Quartile 3, %	22.2	28.3	30.5	28.4
Quartile 4 (highest), %	19.4	32.5	31.8	24.7
**Region**^**b**^				
Urban, %	76.6	82.6	81.4	75.8
Rural, %	23.4	17.4	18.6	24.2
**Marital status**^**b**^				
Married, %	60.2	69.2	70.4	61.4
Divorced/separated/widowed, %	29.2	10.8	7.1	9.5
Never married, %	10.6	20.0	22.5	29.1
**Smoking status**^**b**^				
Never, %	64.4	55.6	56.4	56.6
Former, %	14.8	16.8	16.9	16.8
Current, %	20.9	27.6	26.7	26.6
**Physical Activity (MET-h/wk)**^**b**^				
< 10, %	37.5	31.0	31.8	38.8
≥10, %	62.5	69.0	68.2	61.2
**BMI (kg/m**^**2**^**)**^**b**^				
< 18.5, %	3.1	3.5	5.2	8.3
18.5–22.9, %	40.4	38.0	41.1	44.3
23.0–24.9, %	24.2	24.1	23.1	19.7
≥ 25.0, %	32.3	34.4	30.6	27.7
**Alcohol drinking**^**b**^				
Non-drinkers, %	36.9	21.8	20.3	23.5
glass/day (among drinkers), mean (SD)	1.03 (0.06)	1.09 (0.02)	1.00 (0.01)	0.97 (0.04)
**Self-rated health**^**b**^				
Very good, %	4.6	4.6	5.0	4.2
Good, %	21.5	32.8	35.3	28.8
Fair, %	44.5	47.1	46.6	46.3
Poor, %	29.3	15.4	13.1	20.6
**Metabolic Syndrome**^**b**^				
Present, %	36.3	25.4	22.9	24.1
Absent, %	63.6	74.5	77.0	75.9
Missing, %	0.1	0.1	0.1	0

^a^Weighted percentage and N were estimated by accounting for complex sampling design (strata, cluster, sampling weight)

^b^*P*-value < 0.01. *P*-value was estimated using F test for ANOVA

**Fig. 1:**
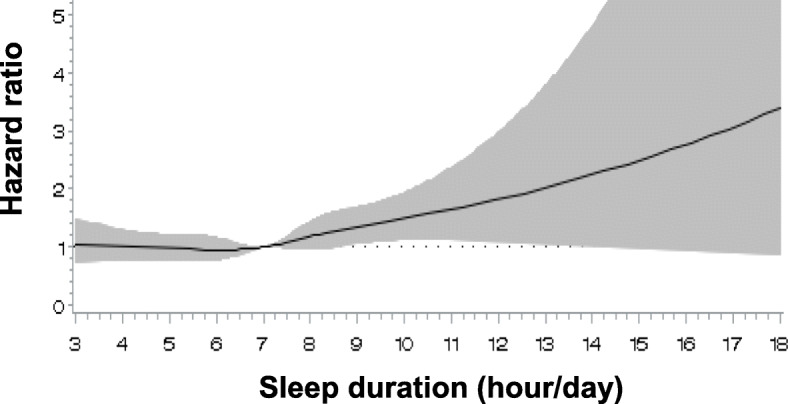
Dose-response relationship of sleep duration with all-cause mortality. This figure shows the results from restricted cubic spline analysis that describes a non-linear relationship between sleep duration and all-cause mortality, adjusting for age (in month, as time metameter), sex (male, female), marital status (married, divorced/separated/widowed, never married), education (less than high school, high school, college or higher), occupation (non-physical labor, physical labor, unemployed), household income (quartiles), region (rural, urban), smoking status (never, former, current), physical activity (< 10, ≥10 MET-h/wk), body mass index (< 18.5, 18.5–22.9, 23.0–24.9, ≥25.0 kg/m^2^), and alcohol drinking (glass/day). Note: Hazard ratios are indicated by solid lines and 95% confidence intervals by shaded areas. Restricted cubic spline models were performed with 5 knots placed at 5th, 35th, 50th, 65th, and 95th percentile of sleep duration (h/day) distribution. A reference point was 7 h/day

Compared with 7–8 h/day of sleep, both short (≤4 h/day: HR = 1.05, 95% CI = 0.79–1.39) and long sleep (≥9 h/day: HR = 1.47, 95% CI = 1.15–1.87) were associated with increased all-cause mortality (Table 2). The lowest all-cause mortality was observed at 5–6 h/day sleep. After additional adjustment for self-rated health, the positive association with short sleep disappeared (HR = 0.99, 95% CI = 0.75–1.32) while the association with long sleep was only slightly attenuated (≥9 h/day: HR = 1.38, 95% CI = 1.08–1.76). After further adjustment for metabolic syndrome, a potential mediator, the associations did not change (≥9 vs. 7–8 h/day: HR = 1.37, 95% CI = 1.08–1.75) (Table 2). Results from spline analysis did not find an evidence for nonlinear relationship between sleep duration and all-cause mortality (p-nonlinearity = 0.26) (Fig. 1). Based on the linear model, every 1-h/day increase in sleep duration was associated with a 7% increased all-cause mortality (95% CI = 1.02–1.12).

**Table 2:** Multivariable (MV)-adjusted hazard ratio (HR) and 95% confidence intervals (CI) for the associations of sleep duration with all-cause and disease-specific mortality

Sleep Duration, h/day	Death/Person-year	Weighted^**a**^ Death/Person-year	HR (95% CI)		
			MV-adjusted^**b**^	MV^**b**^ + self-rated health^**c**^	MV^**b**^ + self-rated health + metabolic syndrome^**d**^
**All-cause mortality**					
**≤ 4**	102/7177	420,860/39,161,266	1.05 (0.79–1.39)	0.99 (0.75–1.32)	1.00 (0.75–1.32)
**5–6**	338/66,585	1,542,062/443,499,465	0.91 (0.76–1.09)	0.90 (0.75–1.08)	0.90 (0.75–1.09)
**7–8**	431/93,979	2,039,248/637,540,363	1.00 (Referent)	1.00 (Referent)	1.00 (Referent)
**≥ 9**	157/13,699	701,830/89,456,218	1.47 (1.15–1.87)	1.38 (1.08–1.76)	1.37 (1.08–1.75)
**Cancer mortality**					
**≤ 4**	29/7177	96,071/39,161,266	0.75 (0.47–1.18)	0.71 (0.45–1.14)	0.72 (0.45–1.14)
**5–6**	119/66,585	500,703/443,499,465	0.83 (0.60–1.14)	0.82 (0.60–1.14)	0.83 (0.60–1.14)
**7–8**	159/93,979	730,251/637,540,363	1.00 (Referent)	1.00 (Referent)	1.00 (Referent)
**≥ 9**	45/13,699	203,146/89,456,218	1.30 (0.86–1.98)	1.24 (0.81–1.89)	1.23 (0.81–1.88)
**Cardiovascular mortality**					
**≤ 4**	18/7177	69,771/39,161,266	0.73 (0.41–1.30)	0.68 (0.38–1.20)	0.67 (0.38–1.20)
**5–6**	72/66,585	359,165/443,499,465	1.04 (0.72–1.50)	1.02 (0.70–1.49)	1.04 (0.71–1.53)
**7–8**	100/93,979	398,680/637,540,363	1.00 (Referent)	1.00 (Referent)	1.00 (Referent)
**≥ 9**	26/13,699	135,659/89,456,218	1.27 (0.73–2.21)	1.19 (0.68–2.06)	1.20 (0.69–2.10)

^a^Weighted deaths and person-years were estimated by accounting for complex sampling design (strata, cluster, sampling weight)

^b^MV-adjusted model included age (in month, as time metameter), sex (male, female), marital status (married, divorced/separated/widowed, never married), education (less than high school, high school, college or higher), occupation (non-physical labor, physical labor, unemployed), household income (quartiles), region (rural, urban), smoking status (never, former, current), physical activity (< 10, ≥10 MET-h/wk), body mass index (< 18.5, 18.5–22.9, 23.0–24.9, ≥25.0 kg/m^2^), and alcohol drinking (glass/day)

^c^Additionally included self-rated health (very good, good, fair, poor)

^d^Additionally included self-rated health (very good, good, fair, poor) and metabolic syndrome (present, absent, missing)

Note: All models were accounted for complex sampling design (sampling weight, cluster, strata)

*Abbreviations*: *CI* confidence interval, *HR* hazard ratio, *MV* multivariable

Table 2 also shows the associations with disease-specific mortality. Long sleep (≥9 vs. 7–8 h/day) was nonsignificantly positively associated with both cancer mortality (HR = 1.30, 95% CI = 0.86–1.98) and CVD mortality (HR = 1.27, 95% CI = 0.73–2.21). After additional adjustment for self-rated health, both associations were attenuated (cancer mortality: HR = 1.24, 95% CI = 0.81–1.89; CVD mortality: HR = 1.19, 95% CI = 0.68–2.06). The positive associations between long sleep and disease-specific mortality did not change after adjustment for metabolic syndrome. Short sleep (≤4 vs. 7–8 h/day) was nonsignificantly inversely associated with both cancer (HR = 0.75, 95% CI = 0.47–1.18) and CVD mortality (HR = 0.73, 95% CI = 0.41–1.30). Results from spline analyses of disease-specific mortality are presented in Supplementary Figure 1 (cancer mortality; *p*-nonlinearity = 0.94) and 2 (CVD mortality; *p*-nonlinearity = 0.79). Although the confidence intervals were wide, the shape of the curves from cancer- and CVD-mortality were similar to that from all-cause mortality, showing an increasing mortality at longer sleep duration.

In subgroup analyses, the overall associations tended to be stronger in younger participants (age < 65 vs. ≥65 years), women (vs. men), and those with college or higher (vs. less than high school) education (Table 3). There was statistically significant positive associations of long sleep with all-cause mortality in participants aged < 65 years (HR = 1.58, 95% CI = 1.00–2.48) and in unemployed participants (HR = 1.52, 95% CI = 1.13–2.04). However, none of the interaction terms were statistically significant (*p*-interaction≥0.39).

**Table 3:** Multivariable-adjusted hazard ratios (HRs) and 95% confidence intervals (CIs) for the associations between sleep duration and all-cause mortality, stratified by demographic and socioeconomic factors

			HR (95% CI)				
	Death/Person-year	Weighted^**a**^ Death/Person-year	Sleep duration, h/day				***P***-interaction
			≤4	5–6	7–8	≥9	
**Age**							
< 65 years	285/148,367	1,773,342/1,085,263,610	1.46 (0.75–2.83)	0.87 (0.64–1.20)	1.00 (Referent)	1.58 (1.00–2.48)	0.56
≥ 65 years	743/33,073	2,930,659/124,393,702	0.86 (0.64–1.15)	0.87 (0.71–1.07)	1.00 (Referent)	1.27 (0.97–1.67)	
**Sex**							
Male	620/77,615	2,913,160/611,437,975	1.06 (0.69–1.64)	0.99 (0.79–1.24)	1.00 (Referent)	1.35 (0.96–1.89)	0.69
Female	408/103,824	1,790,841/598,219,336	0.97 (0.66–1.45)	0.78 (0.59–1.03)	1.00 (Referent)	1.43 (0.99–2.06)	
**Education**							
Less than High school	782/48,304	3,343,865/317,448,992	0.94 (0.71–1.26)	0.92 (0.74–1.14)	1.00 (Referent)	1.39 (1.06–1.81)	0.89
High school	128/35,469	963,827/489,412,883	1.20 (0.45–3.23)	0.83 (0.55–1.28)	1.00 (Referent)	1.23 (0.63–2.39)	
College or higher	118/97,668	396,310/ 402,795,436	2.02 (0.61–6.69)	0.87 (0.50–1.54)	1.00 (Referent)	1.75 (0.62–4.95)	
**Occupation**							
Non-physical labor	47/38,660	269,418/293,918,617	2.36 (0.42–3.36)	0.69 (0.37–1.31)	1.00 (Referent)	0.43 (0.04–4.42)	0.39
Physical labor	357/73,971	1,662,546/494,418,683	1.25 (0.71–2.20)	0.92 (0.67–1.26)	1.00 (Referent)	1.16 (0.73–1.84)	
Unemployed	624/68,809	2,772,037/421,320,011	0.92 (0.67–1.27)	0.89 (0.72–1.12)	1.00 (Referent)	1.52 (1.13–2.04)	

^a^Weighted deaths and person-years were estimated by accounting for complex sampling design (strata, cluster, sampling weight)

All models were accounted for complex sampling design (sampling weight, cluster, strata) and included age (in month, as time metameter), sex (male, female), marital status (married, divorced/separated/widowed, never married), education (less than high school, high school, college or higher), occupation (non-physical labor, physical labor, unemployed), household income (quartiles), region (rural, urban), smoking status (never, former, current), physical activity (< 10, ≥10 MET-h/wk), body mass index (< 18.5, 18.5–22.9, 23.0–24.9, ≥25.0 kg/m^2^), alcohol drinking (glass/day), and self-rated health (very good, good, fair, poor)

*P*-interaction was estimated using Wald test for product terms

*Abbreviations*: *CI* confidence interval, *HR* hazard ratio, *MV* multivariable

In sensitivity analyses, similar results were found after excluding deaths occurred during the first 2 years of follow-up (with time-lagged analysis) and after restricting to those with ≥5 years of follow-up (data not shown).

## Discussion

In this study, we observed the lowest mortality at 5–6 h/day sleep. Compared with 7–8 h/day of sleep, short (≤4 h/day) and long (≥9 h/day) sleep were associated with a 5% and 47% increased all-cause mortality, respectively. Our findings are consistent with previous studies that reported increased mortality associated with both short and long sleep [14, 17, 22, 29–31]. However, the risk estimates from our study are somewhat smaller for short sleep but larger for long sleep than those from another study conducted in Koreans with longer follow-up (average follow-up of 9.4 years; 21% and 36% increased mortality associated with ≤5 and ≥ 10 vs. 7 h/day, respectively) [22]. Possible explanations for the variation in magnitude of associations across studies are the differences in length of follow-up, population characteristics (e.g., age, genetic predisposition), and methodological issues (e.g., inadequate control for confounding). In our study, we excluded participants with a prior diagnosis of CVD or cancer at baseline to reduce confounding by preexisting diseases. We also observed that the associations were attenuated after adjustment for self-rated health, including disappearance of positive association with short sleep, suggesting that the sleep-mortality association can be overestimated in studies that failed to control for subclinical diseases. Some health conditions may influence individual’s quantity and quality of sleep years before they can be clinically diagnosed. Similarly, other studies have also shown the attenuation in associations after excluding individuals with poor health conditions at baseline [2, 18, 32].

In the present study, we observed a stronger positive association with long sleep than with short sleep. In a previous meta-analysis [17], Asian cohorts showed higher mortality risk associated with long sleep compared with those from other populations, suggesting that long sleep may be more harmful in Asians. When we stratified by age in the present study, in older population (aged ≥65 years) the positive association was found with long sleep only, whereas in younger population (< 65 years) both short and long sleep were positively associated with mortality. Consistently, other studies of elderly population found a positive association with long sleep and no association with short sleep [2, 18, 33], whereas a study that was conducted among middle-aged men in Korea found a positive association with short sleep (≤5 h/day) only [34]. The lack of positive association with short sleep in older population (in contrast to younger population) may be possibly due to the physiological differences that requires shorter sleep in older adults as a natural process of aging [35, 36]. The optimal sleep duration may vary by age. Because participants were not explicitly asked to include nap time in their reports of daily sleep duration, it is also possible that participants who reported to have short sleep duration may already have adequate sleep from a nap, contributing to underestimation of positive association between short sleep and mortality in our study. As nap time may vary by age and differentially influence mortality [37], further studies are needed to investigate the differences in associations between younger and older adults after accounting for nap time. Further, studies also suggested that the associations with sleep duration may vary by sex. Consistent with prior studies, we observed stronger associations in women (43% vs. 35% increased mortality associated with long sleep in women vs. men) [22, 38]. However, little is known about the sex differences in optimal sleep duration. Further studies are needed to confirm our results and clarify the biological underpinnings of sex differences.

When we examined the associations by causes of death, long sleep was associated with a nonsignificant 30% increased cancer mortality, similar to the findings from another Korean cohort [22]. Other studies from Asian cohorts (Chinese, Japanese) also showed an increased cancer mortality at ≥10 h/day sleep [18, 38]. However, a study from the US did not find an association with cancer mortality [30]. Because sleep duration may be differentially associated with specific cancer types [9], the variation in results across geographic regions may be partly due to the distribution of specific cancer deaths in the population. Given the limited number of cancer deaths, we were not able to investigate the associations by different cancer types in the present study. With CVD mortality, we also observed a similar nonsignificant positive association (27% increased CVD mortality) with long sleep. Our findings somewhat disagree with previous studies [18, 22, 30, 38, 39] which reported an increased CVD mortality associated with both short and long sleep. As a previous meta-analysis found a positive association with short sleep when specific CVD type (stroke, coronary heart disease) was examined but not when total CVD was examined [40], the association with short sleep may be specific to certain CVD type. Because we had limited number of disease-specific deaths, it is also possible that we did not have sufficient power to detect differences in disease-specific mortality.

There are several biological mechanisms that could explain the association between sleep duration and mortality. Suboptimal sleep duration may increase the risk of CVD, obesity, and diabetes though inflicting physiologic stress (neuroendocrine stress system) and influencing immune system [4, 11]. Sleep patterns can also influence brain responses to secrete proinflammatory cytokines [1]. Sleep deprivation also alters glucose metabolism and decreases energy expenditure [6], leading to an increased risk of obesity and diabetes. The biological mechanisms of long sleep are yet unclear but there are many potential pathways including sleep fragmentation, fatigue, impaired immune function, inflammation, photoperiodic abnormalities, depression, and an increase risk of heart disease and frailty [33, 41, 42]. In our study, we observed that the positive association between long sleep and mortality persisted after adjustment for metabolic syndrome, supporting the role of other mechanisms that are not related to metabolic syndrome and the related cardiometabolic profiles. Long sleepers inevitably have a shorter awake time, decreasing chance to engage in an activity with beneficial effects (e.g., exercise), and therefore may harbor poor health conditions [41]. In our data, long sleepers were also less likely to meet the physical activity guidelines than average sleepers. Further, long sleep may also indicate poor sleep quality such as sleep fragmentation [41]. In many cohorts including ours, data on sleep quality as well as sleep disorders were not available and thus further studies are needed to investigate the role of sleep quality in the relationship with mortality.

We acknowledge several limitations of this study. First, sleep duration was self-reported via health interview and thus our exposure data are prone to measurement error. However, a previous validation study reported a moderate correlation (*r* = 0.47) between self-reported and measured sleep duration [43] and, in our prospective analysis, the measurement error is likely to be non-differential, possibly contributing to underestimation of the association [18]. Previous studies that measured sleep duration using actigraph (accelerometer) found similar results but the lowest mortality was observed at a shorter sleep than those reported from the self-reported data [16]. Second, we used the exposure data on average duration of sleep during the past year. Due to lack of data, we were not able to examine the influence of nap time, as well as the differences between weekday vs. weekend sleep duration. Our survey also did not collect information on sleep quality [44] and sleep disorder [45] and thus we were not able to account for these variables in our analysis. Individuals with sleep disorder such as sleep apnea and insomnia are more likely to have shorter sleep duration [46, 47] and higher risk of all-cause mortality [46–50]. Therefore, failure to take account of diagnosis and treatment of sleep disorder may lead to overestimation of positive association between short sleep and mortality. In our study, we adjusted for self-rated health to reduce bias due to any health-related sleep deprivation and observed that the association with short sleep disappeared after the adjustment. Other studies also suggested an attenuated association between short sleep and mortality after accounting for sleep quality, use of hypnotics [31], and insomnia [51]. Third, as the survey was conducted once at baseline, we were not able to account for changes in exposure and covariate status during the follow-up. Lastly, our study included an average of 6.3 years of follow-up (up to 9.5 years). With limited follow-up and number of deaths, some stratified analyses did not have sufficient power to detect a small difference.

Despite the limitations, there are several strengths in our study. First, using a prospective study design, we reduced the possibility of recall bias. Second, we reduced possible bias due to preexisting diseases by excluding individuals with a history of CVD or cancer at baseline, excluding individuals who died during the first 2 years of follow-up (with time-lagged analysis), and additionally adjusting for poor self-rated health. In these sensitivity analyses, our results were shown robust. Third, we reduced confounding by careful adjustment for a range of covariates, including demographic characteristics and lifestyle factors. Lastly, our study population was a nationally representative sample of Korean adults and thus our results are likely to be generalizable to the general Korean population.

## Conclusion

In summary, our study found a positive association between long sleep and all-cause mortality in Korean adults. While obesity, physical inactivity, alcohol drinking, and smoking have been the main focus of many behavioral interventions, our findings suggest that sleep duration may be another risk factor that needs to be targeted for effective prevention of chronic diseases and premature deaths. However, given our limited sample size and follow-up, further studies are needed to confirm our results. Additionally, potential confounders such as sleep quality and daytime sleep should be considered in future studies.

## Supplementary Information

**Additional file 1: Supplementary Figure 1.** Non-linear relationship of sleep duration with cancer mortality. This figure shows the results from spline analysis that describes a non-linear relationship between sleep duration and cancer mortality, adjusting for age (in month, as time metameter), sex (male, female), marital status (married, divorced/separated/widowed, never married), education (less than high school, high school, college or higher), occupation (non-physical labor, physical labor, unemployed), household income (quartiles), region (rural, urban), smoking status (never, former, current), physical activity (< 10, ≥10 MET-h/wk), body mass index (< 18.5, 18.5–22.9, 23.0–24.9, ≥25.0 kg/m^2^), and alcohol drinking (glass/day).

**Additional file 2: Supplementary Figure 2.** Non-linear relationship of sleep duration with CVD mortality. This figure shows the results from spline analysis that describes a non-linear relationship between sleep duration and cancer mortality, adjusting for age (in month, as time metameter), sex (male, female), marital status (married, divorced/separated/widowed, never married), education (less than high school, high school, college or higher), occupation (non-physical labor, physical labor, unemployed), household income (quartiles), region (rural, urban), smoking status (never, former, current), physical activity (< 10, ≥10 MET-h/wk), body mass index (< 18.5, 18.5–22.9, 23.0–24.9, ≥25.0 kg/m^2^), and alcohol drinking (glass/day).

## Data Availability

The datasets supporting the conclusions of this article are available from the KCDC on reasonable request. The KNHANES datasets and corresponding survey questionnaires are publicly available at https://knhanes.cdc.go.kr . The 「Korea National Health and Nutrition Examination Survey linked Cause of death data」 are available with permission of KCDC.

## Abbreviations

BMI: Body mass index
CI: Confidence interval
CVD: Cardiovascular disease
HDL: High-density lipoprotein
HR: Hazard ratio
KCDC: Korea Centers for Disease Control and Prevention
KNHANES: Korea National Health and Nutrition Examination Survey
MET: Metabolic equivalent task
