# Supplementary material for: Sleep duration and mortality in Korean adults: a population-based prospective cohort study
Source: BMC Public Health. 2020 Oct 28;20:1623. doi: 10.1186/s12889-020-09720-3 (PMC7594310; doi:10.1186/s12889-020-09720-3)
Supplement: Supplementary file 1 — Additional file 1: Supplementary Figure 1. Non-linear relationship of sleep duration with cancer mortality. This figure shows the results from spline analysis that describes a non-linear relationship between sleep duration and cancer mortality, adjusting for age (in month, as time metameter), sex (male, female), marital status (married, divorced/separated/widowed, never married), education (less than high school, high school, college or higher), occupation (non-physical labor, physical labor, unemployed), household income (quartiles), region (rural, urban), smoking status (never, former, current), physical activity (< 10, ≥10 MET-h/wk), body mass index (< 18.5, 18.5–22.9, 23.0–24.9, ≥25.0 kg/m2), and alcohol drinking (glass/day). [file 12889_2020_9720_MOESM1_ESM.pptx]

## Slide 1
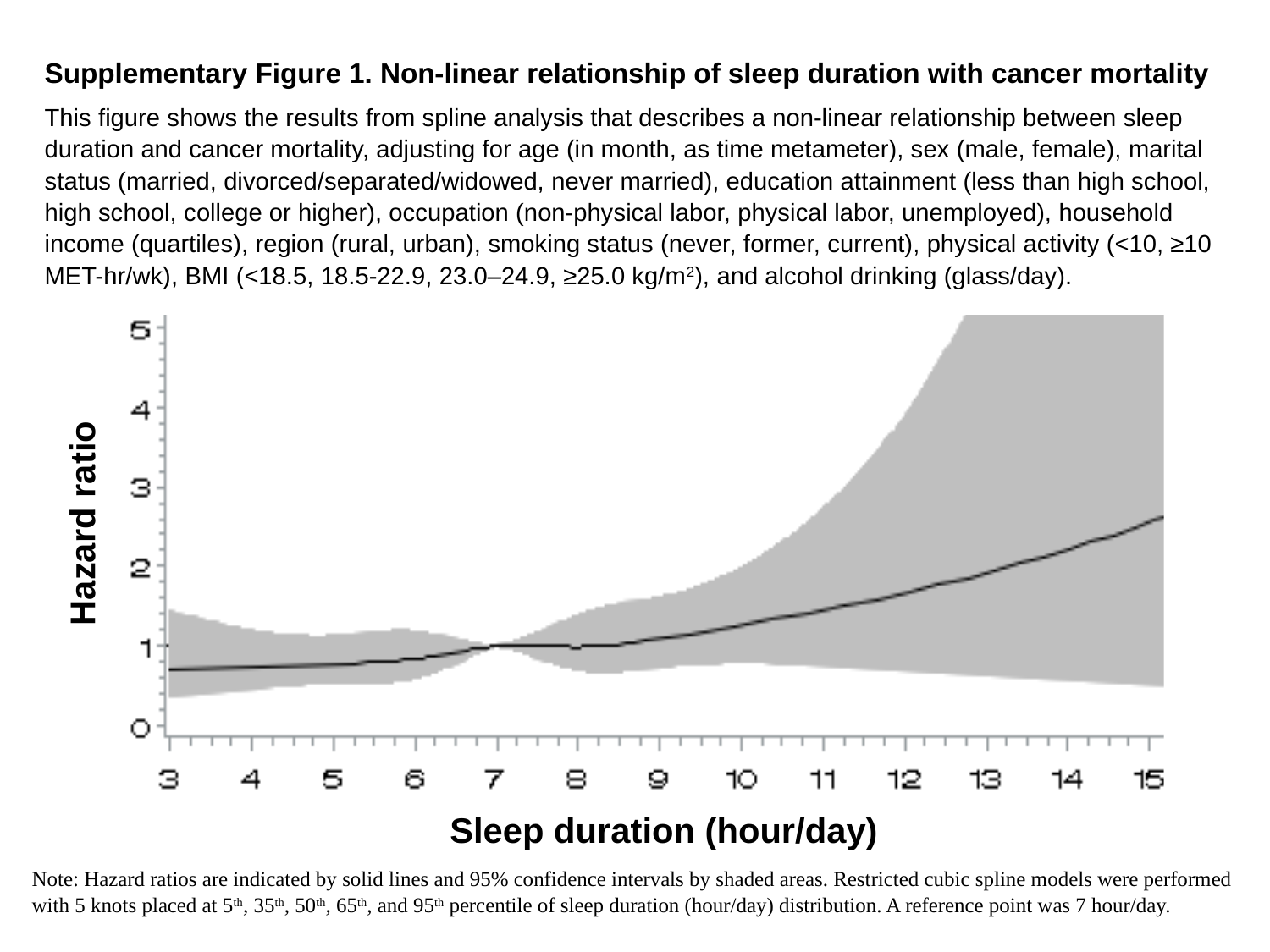

Supplementary Figure 1. Non-linear relationship of sleep duration with cancer mortality
This figure shows the results from spline analysis that describes a non-linear relationship between sleep duration and cancer mortality, adjusting for age (in month, as time metameter), sex (male, female), marital status (married, divorced/separated/widowed, never married), education attainment (less than high school, high school, college or higher), occupation (non-physical labor, physical labor, unemployed), household income (quartiles), region (rural, urban), smoking status (never, former, current), physical activity (<10, ≥10 MET-hr/wk), BMI (<18.5, 18.5-22.9, 23.0–24.9, ≥25.0 kg/m2), and alcohol drinking (glass/day).
Hazard ratio
Sleep duration (hour/day)
Note: Hazard ratios are indicated by solid lines and 95% confidence intervals by shaded areas. Restricted cubic spline models were performed with 5 knots placed at 5th, 35th, 50th, 65th, and 95th percentile of sleep duration (hour/day) distribution. A reference point was 7 hour/day.
